# 

**Published:** 1890-12

**Authors:** 


					DECEMBER, 1890.
Vo1' VIII. No. 30.
THE
BRISTOL
medico-chirurgical
r *
fj
' ? \
? \T
\ "
yA
JOURNAL
B <&uarterl? journal of tbe jflRc&ical Sciences for tbe vilest
of 3Englan& attD Soutb "CGlales
PUBLISHED UNDER THE AUSPICES OF
THE BRISTOL MEDICO-CHIRURGICAL SOCIETT.
EDITOR:
J. GREIG SMITH, M.A., F.R.S.E.
ASSISTANT-EDITOR : y?___ ^
L. M. GRIFFITHS.
"Scire eat nescire, nfeUa me
Scire alius aclret."
BRISTOL: J. W. ARROWSMITH-,
London: J. & A. Churchill, 11 New Bttrt-ington Street.
Price One Shilling and Sixpence.

				

